# Annual Carbon Emissions from Deforestation in the Amazon Basin between 2000 and 2010

**DOI:** 10.1371/journal.pone.0126754

**Published:** 2015-05-07

**Authors:** Xiao-Peng Song, Chengquan Huang, Sassan S. Saatchi, Matthew C. Hansen, John R. Townshend

**Affiliations:** 1 Department of Geographical Sciences, University of Maryland, College Park, Maryland, United States of America; 2 Global Land Cover Facility, University of Maryland, College Park, Maryland, United States of America; 3 Jet Propulsion Laboratory, California Institute of Technology, Pasadena, California, United States of America; Cirad, FRANCE

## Abstract

Reducing emissions from deforestation and forest degradation (REDD+) is considered one of the most cost-effective strategies for mitigating climate change. However, historical deforestation and emission rates―critical inputs for setting reference emission levels for REDD+―are poorly understood. Here we use multi-source, time-series satellite data to quantify carbon emissions from deforestation in the Amazon basin on a year-to-year basis between 2000 and 2010. We first derive annual deforestation indicators by using the Moderate Resolution Imaging Spectroradiometer Vegetation Continuous Fields (MODIS VCF) product. MODIS indicators are calibrated by using a large sample of Landsat data to generate accurate deforestation rates, which are subsequently combined with a spatially explicit biomass dataset to calculate committed annual carbon emissions. Across the study area, the average deforestation and associated carbon emissions were estimated to be 1.59 ± 0.25 M ha•yr^−1^ and 0.18 ± 0.07 Pg C•yr^−1^ respectively, with substantially different trends and inter-annual variability in different regions. Deforestation in the Brazilian Amazon increased between 2001 and 2004 and declined substantially afterwards, whereas deforestation in the Bolivian Amazon, the Colombian Amazon, and the Peruvian Amazon increased over the study period. The average carbon density of lost forests after 2005 was 130 Mg C•ha^−1^, ~11% lower than the average carbon density of remaining forests in year 2010 (144 Mg C•ha^−1^). Moreover, the average carbon density of cleared forests increased at a rate of 7 Mg C•ha^−1^•yr^−1^ from 2005 to 2010, suggesting that deforestation has been progressively encroaching into high-biomass lands in the Amazon basin. Spatially explicit, annual deforestation and emission estimates like the ones derived in this study are useful for setting baselines for REDD+ and other emission mitigation programs, and for evaluating the performance of such efforts.

## Introduction

Deforestation is considered the second largest anthropogenic source of carbon dioxide to the atmosphere [1]. While annual carbon emissions from fossil-fuel combustion have been continually increasing since 1960s, historical trends of deforestation and associated carbon emissions have remained poorly understood [1–3]. Using various data and methods recent studies estimate that deforestation in the tropics accounts for 0.6 to 2.0 Pg C yr^-1^ of the carbon emitted to the atmosphere in the 1980s, 0.9 to 2.2 Pg C yr^-1^ in the 1990s, and 0.8 to 2.9 Pg C yr^-1^ in the 2000s [4–9]. The large range of emission estimates is due to high uncertainty in quantifying deforestation rates, differences in the definition of forest loss, and the methodology used to estimate emission intensity.

The United Nations Food and Agriculture Organization (FAO) provide periodic update on net changes in the area and biomass of the world’s forests at about 5- or 10-year intervals mainly based on country reporting, with supplementary satellite sample analysis. Centralizing information through country participation is valuable in some aspects and the resulting national statistics have been widely used in a number of scientific applications [9–12]. However, limitations of FAO’s Global Forest Resource Assessment are also discussed in the literature, including primarily the lack of internal consistency due to different definitions of forest among countries and time intervals [2, 13]. Additionally, reporting forest area change as land-use change does not reflect the biophysical consequences of land surface transformation [14]. Furthermore, only reporting net changes in forest area, without partition to gross loss and gain, could lead to ambiguous target (e.g. “zero deforestation”) for current and future deforestation mitigation programs [15].

Satellite-based observations of forest cover change provide an alternative to estimate deforestation rates consistently across space and time. At continental to global scales, maps of forest cover and change are increasingly being generated from various satellite data sources. Among the latest progresses, Landsat samples have been used to determine tropical deforestation rates between 1990 and 2010 [16]; Moderate Resolution Imaging Spectroradiometer (MODIS) and Landsat data have been jointly used to quantify global gross forest cover loss between 2000 and 2005 [17, 18]; wall-to-wall Landsat 5 Thematic Mapper (TM) and Landsat 7 Enhanced Thematic Mapper Plus (ETM+) surface reflectance data have been used to derive global percent tree cover maps for circa 2000 and 2005 [19] with change maps between 1975, 1990, 2000 and 2005 being generated [20]; Landsat ETM+ top-of-atmosphere (TOA) reflectance data have been composited at annual resolution to create global forest cover loss and gain maps between 2000 and 2012 [14]; the Advanced Land Observing Satellite Phased Array L-band Synthetic Aperture Radar data have been employed to produce forest/non-forest maps in Southeast Asia [21] as well as over the globe [22].

Some of these forest cover change datasets have been integrated with satellite-based forest biomass information [6, 23] to quantify changes in forest carbon stocks [8, 16, 24]. These existing studies clearly reveal the spatial heterogeneity of land-cover change emissions across ecological and/or political boundaries. However, an average emission estimate over a 5- or 10-year interval, similar to FAO’s reports, does not embrace the necessary temporal details to uncover historical trends. Unlike fossil-fuel emissions that are known to have been increasing steadily [1, 3], a limited number of studies suggest that forest cover change rates can fluctuate substantially from year to year [14, 25, 26]. The temporal variability of deforestation at continental to global scales has yet to be understood and linked to the global carbon cycle.

Quantifying trends and temporal variability of carbon emissions from deforestation is important for a number of reasons. First, it may explain some of the inter-annual variability of atmospheric CO_2_ concentration. Atmospheric inversion studies suggest that the inter-annual variability of global CO_2_ growth rate is dominated by tropical land ecosystems, with positive anomalies related to El Niño and negative anomalies related to La Niña [27–29]. A recent study further recognizes that semi-arid ecosystems may become a more relevant driver of the global carbon anomaly in the future [30], but questions remain about how much of the variability can be attributed to carbon released from land-cover change [31]. Second, the trend of deforestation is critical for understanding the complex and changing drivers of deforestation [32]. For example, the increasing deforestation between 2001 and 2004 in the Brazilian Amazon is related to trends in the international soybean price and the declining deforestation after 2005 is associated with the collapse of commodity markets as well as shifting land-use dynamics [33, 34]. Studies also link time-series deforestation emissions with economic input-output models to attribute emissions to domestic consumption and to international trade of agricultural products [35]. Third, knowing the trend and variability of historical emissions likely has a strong influence on policies of reducing emissions from deforestation and forest degradation (REDD+). The inter-annual variability itself is a key variable for setting reference emission level (REL) or baseline in some proposed REL methods (e.g. the corridor approach [36]).

The objective of this study is to quantify annual deforestation and related carbon emissions using time-series satellite data. Our study area is the Amazon basin, which occupies about 40% of South America. More than 60% of the basin is located in Brazil and the rest in Bolivia, Colombia, Ecuador, French Guiana, Guyana, Peru, Suriname and Venezuela. Most of the basin is covered by closed canopy rainforests, which provide habitat for a vast array of plant and animal species [37]. The leading environmental issue in this region is the pervasive loss of pristine forests, threatening terrestrial biodiversity [38] and altering regional and global climate [39]. Deforestation is driven by a variety of complex socioeconomic and natural factors [32], including mechanized agricultural expansion (cattle ranching and soybean plantation) in the Brazilian Amazon [33, 34], illegal plantation (e.g. coca) in the Colombian Amazon [40], gold mining in the Peruvian Amazon [41], as well as droughts, fires and floods in many different places of the basin [42–44].

For this study, forest refers to an area of at least 0.09 ha in size that is covered by 25% or more trees that are 5 m or taller. Consistent with others [8], deforestation is defined as the reduction of tree cover to below 25%. Annual deforestation rates are generated using yearly tree cover maps derived at 250 m resolution from the MODIS vegetation continuous field (VCF) product [45] and then calibrated using a large sample of 30 m Landsat images, which more reliably depict change. We then combine the deforestation rates with a circa 2000 forest biomass dataset [23] to quantify annual carbon emissions from deforestation by applying the standard methodology described in [8]. Our estimates include deforestation due to all causes including wildfires, flooding and anthropogenic clearing. Following suggestions by [15], we estimate carbon fluxes from gross deforestation without the inclusion of forest regrowth in order to inform ongoing policy discussions on REDD+, which is also consistent with recent studies [8, 24]. Our emission estimates include loss of above and below ground biomass in the deforested area. Changes in the soil carbon pool due to deforestation are not included.

## Results

### Annual Deforestation Rates in the Amazon Basin

**Fig 1:**
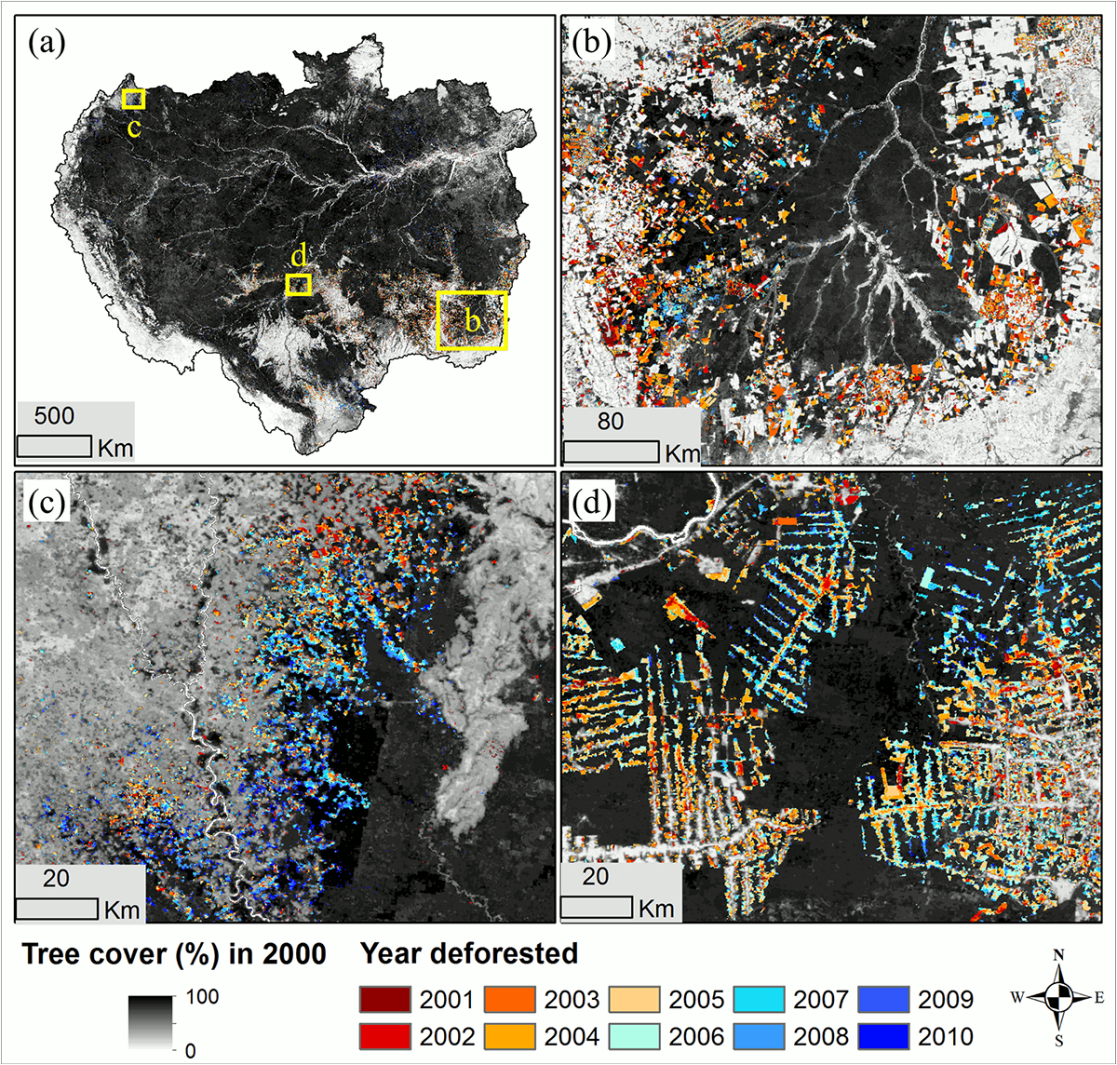
Deforestation year map derived from time-series of MODIS VCF tree cover dataset. (a) Overview of the Amazon basin with yellow boxes indicating the locations of regional close-ups. (b) Close-up over the Xingu river basin in Mato Grosso, Brazil. The large patch of remaining intact forest is a consequence of protection status in this area. (c) Close-up in Colombia. (d) Close-up in Rondonia, Brazil.

The deforestation map products derived through this study identified the year of forest clearing for every MODIS pixel within the Amazon basin (Fig 1). Between 2000 and 2010, a total of 15.9 ± 2.5 M ha (million ha) forests were lost, which represented 2.6% of the total basin area, or 2.9% of forests in year 2000. The Brazilian Amazon and the non-Brazilian Amazon lost a total of 12.5 ± 2.0 M ha and 3.4 ± 0.5 M ha forests respectively over that decade. Brazil was the dominant country in terms of deforested area, which accounted for 79% of the total lost forests. Following Brazil, Bolivia contributed the second most deforestation in the last decade, which accounted for 12% (1,969 ± 212 K ha) of the basin total, more than the sum of the Peruvian Amazon (6%, or 979 ± 123 K ha) and the Colombian Amazon (2%, or 287 ± 67 K ha).

**Fig 2:**
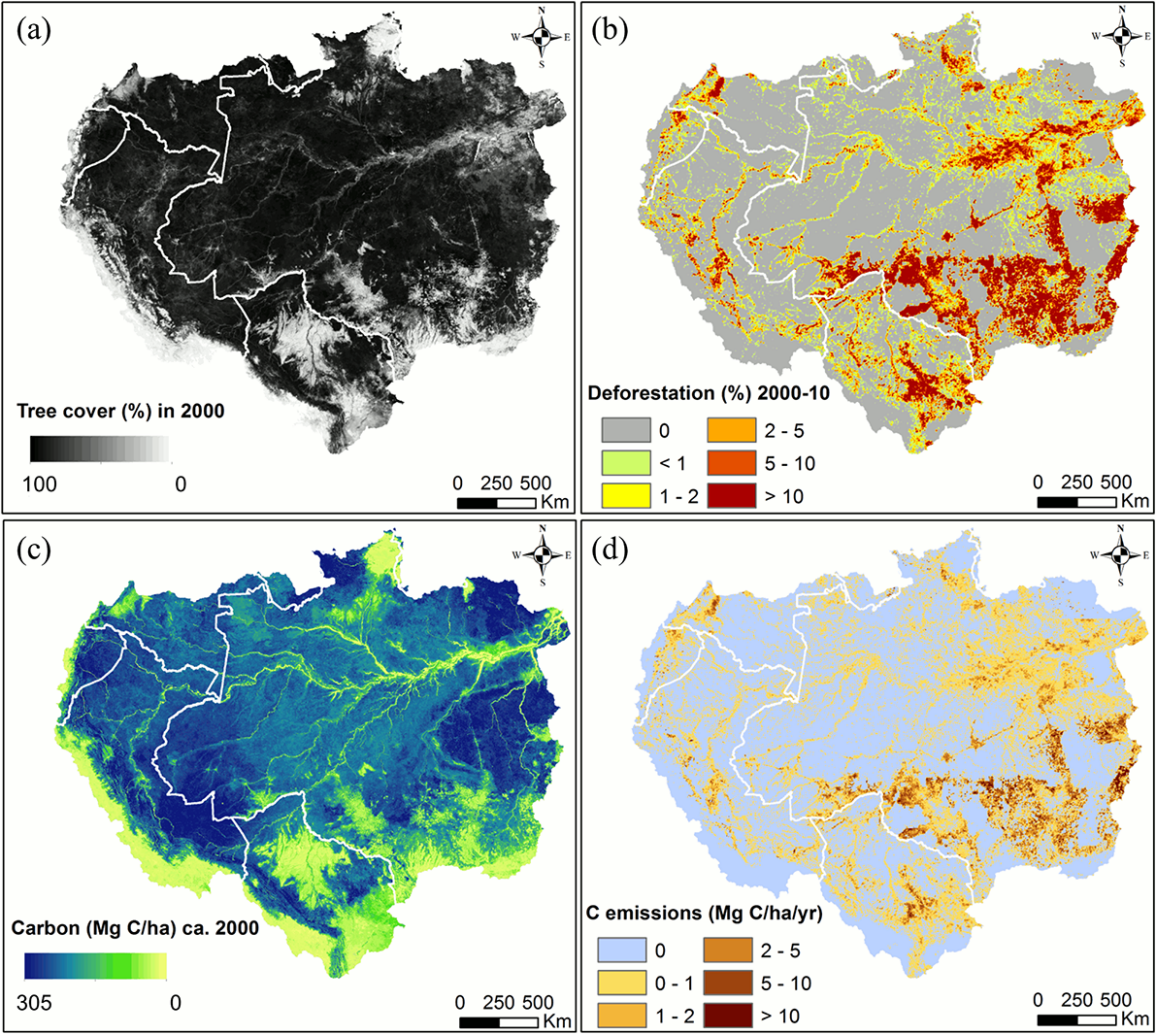
Maps of forest, deforestation, carbon stocks and carbon emissions in the Amazon basin. (a) Tree cover in year 2000 (b) Deforestation between 2000 and 2010 at 5 km spatial resolution. (c) Forest carbon density circa 2000. (d) Average C emission rate per unit deforestation at 5 km spatial resolution. White lines delineate country boundaries.

**Fig 3:**
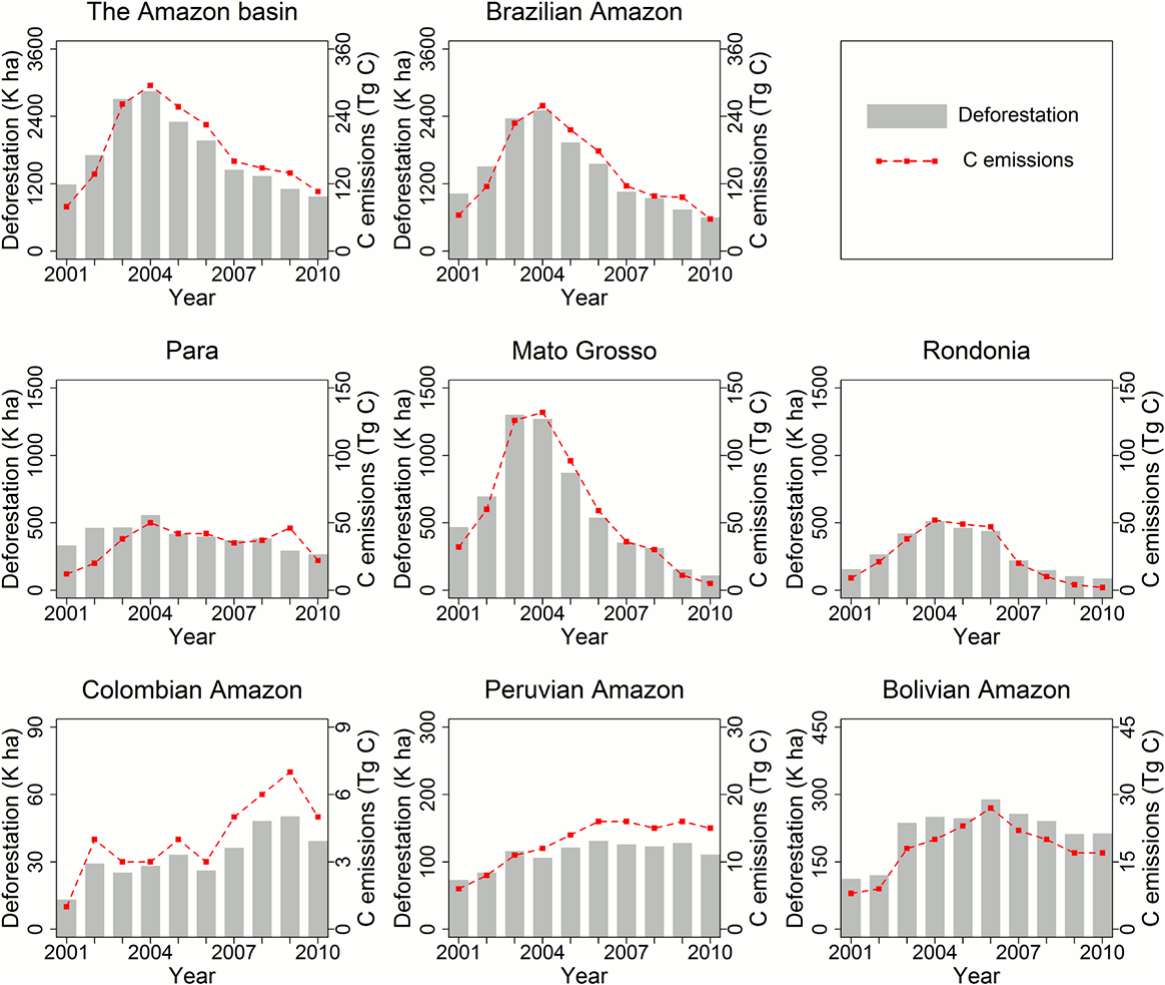
Trends of deforestation and associated carbon emissions from 2000 to 2010.

The geographic locations of deforestation were largely concentrated on the southeastern edge of the basin (the so called “arc of deforestation”), with new hotspots emerging in western Amazon (Fig 2). Consistent with reports by the Brazilian government, the FAO and other previous studies [14, 46–48], a declining trend in the Brazilian Amazon and the entire Amazon basin after 2005 was confirmed (Fig 3). The annual relative share of Brazil’s deforestation changed dramatically over the study period−from the highest of 87% in the year 2004 to the lowest of 54% by the year 2010. The largest decline in deforestation rate was observed in Mato Grosso, from 1,200 K ha in 2004 to below 100 K ha in 2010. Obvious declines were also observed in Rondonia and Para, though to lesser degrees. These three states accounted for more than 80% of forest clearing in Brazil. In the western and southern parts of the basin, deforestation rates in the Peruvian Amazon and the Bolivian Amazon also decreased slightly after 2006. In the Colombian Amazon, annual rates nearly doubled from 2006 to 2009, although the total area cleared there was much lower than those in the other countries or states.

**Fig 4:**
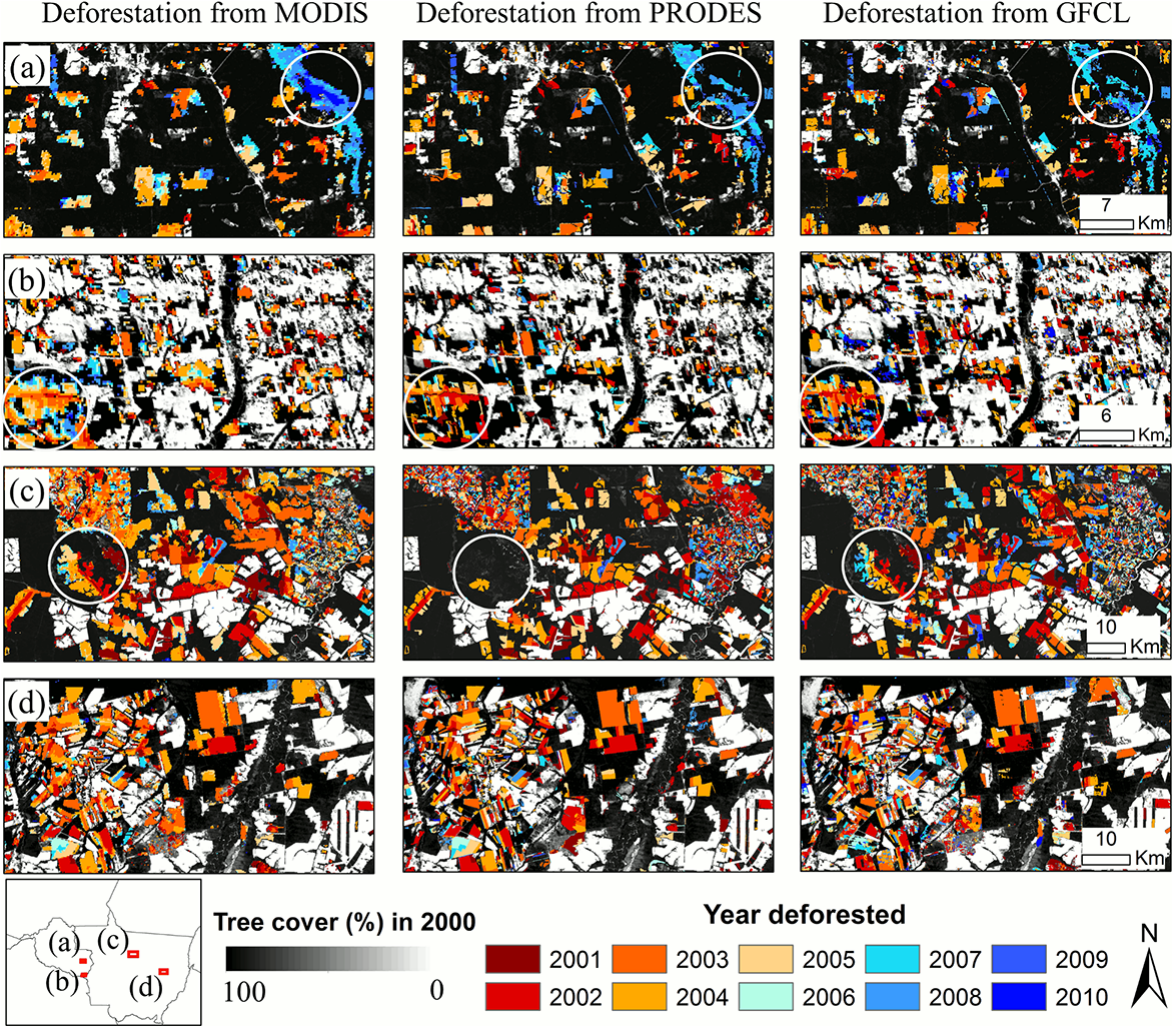
Comparing annual deforestation derived from MODIS with Landsat-based maps in the Brazilian Amazon. The left column is MODIS results from this study, the middle column is PRODES (Program for the Annual Estimation of Deforestation in the Brazilian Amazon) and the right column is GFCL (Global Forest Cover Loss). The spatiotemporal patterns of deforestation agree remarkably well in these products with some disagreement highlighted in circles. (a) Deforestation near Pimenta Bueno, Rondonia (60.821W, 12.088S); (b) Deforestation to the south of Colorado do Oeste in Rondonia (60.754W, 13.396S); (c) Forests cleared for agriculture in central Mato Gross (55.974W, 11.466S); (d) Deforestation to the south of indigenous reserves in the lower Xingu river basin in Mato Grosso (53.033W, 13.158S). The MODIS map likely omits cleared patches below the 250 m pixel size and it also tends to overestimate deforestation over small but inter-connected patches (b). The two Landsat-based products have an advantage to real small-patch clearings, but may overlook some deforestation due to missing data from e.g. cloud contamination (a and c).

**Fig 5:**
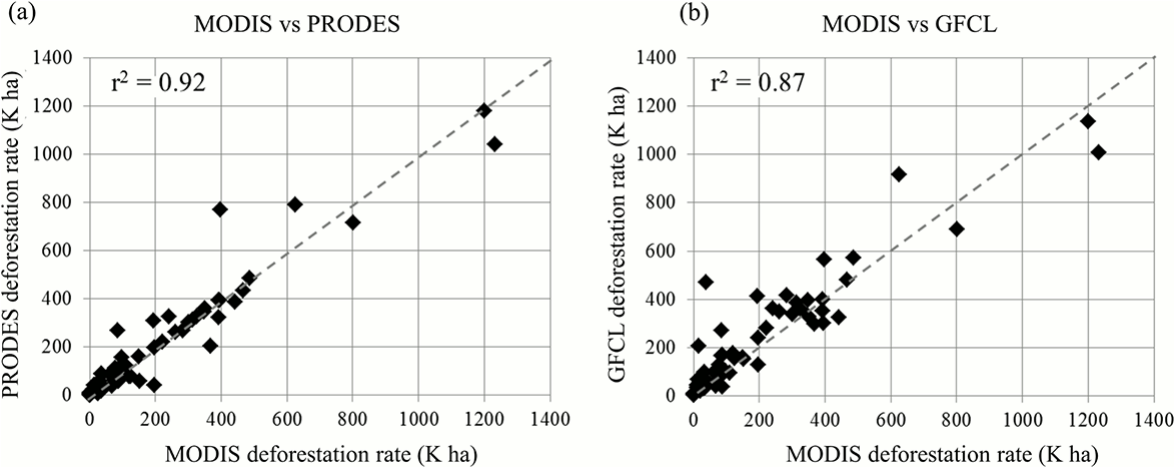
Comparing MODIS-derived annual deforestation rates with Landsat results in the Brazilian Amazon. (a) MODIS vs PRODES. (b) MODIS vs GFCL. A total of 70 data points are used in the scatter plot, which represent annual estimates between 2000 and 2010 in seven Brazilian states in the legal Amazon, including Acre, Amapa, Amazonas, Mato Grosso, Para, Rondonia and Roraima.

Deforestation estimates derived through this study were comparable to those derived based on Landsat data. At individual patch level, the deforestation maps derived through this study had spatiotemporal patterns similar to the PRODES (Program for the Annual Estimation of Deforestation in the Brazilian Amazon) product [48] and a Landsat-based global forest cover loss (GFCL) dataset [14] (Fig 4). At the state-level, annual deforestation rates derived through this study were highly correlated with those calculated based on the two Landsat-based products (Fig 5). Over the Brazilian Amazon basin, the total deforestation rates over the 11-year period derived based on PRODES (12.8 M ha) and GFCL (14.6 M ha) were within or near the upper bound of our estimate.

### Annual Gross Carbon Emissions from Deforestation in the Amazon Basin

Assuming immediate carbon release at forest clearing [8, 16], the total committed carbon emissions due to loss of above and below ground biomass within the Amazon basin were estimated to be 1.81 ± 0.68 Pg C between 2000 and 2010, or 0.18 ± 0.07 Pg C yr^-1^. Not surprisingly, the largest share of emissions was found in Brazil (79%, 143 ± 56 Tg C yr^-1^), followed by Bolivia (10%, 18 ± 8 Tg C yr^-1^), Peru (7%, 13 ± 3 Tg C yr^-1^) and Colombia (2%, 4 ± 1 Tg C yr^-1^).

**Fig 6:**
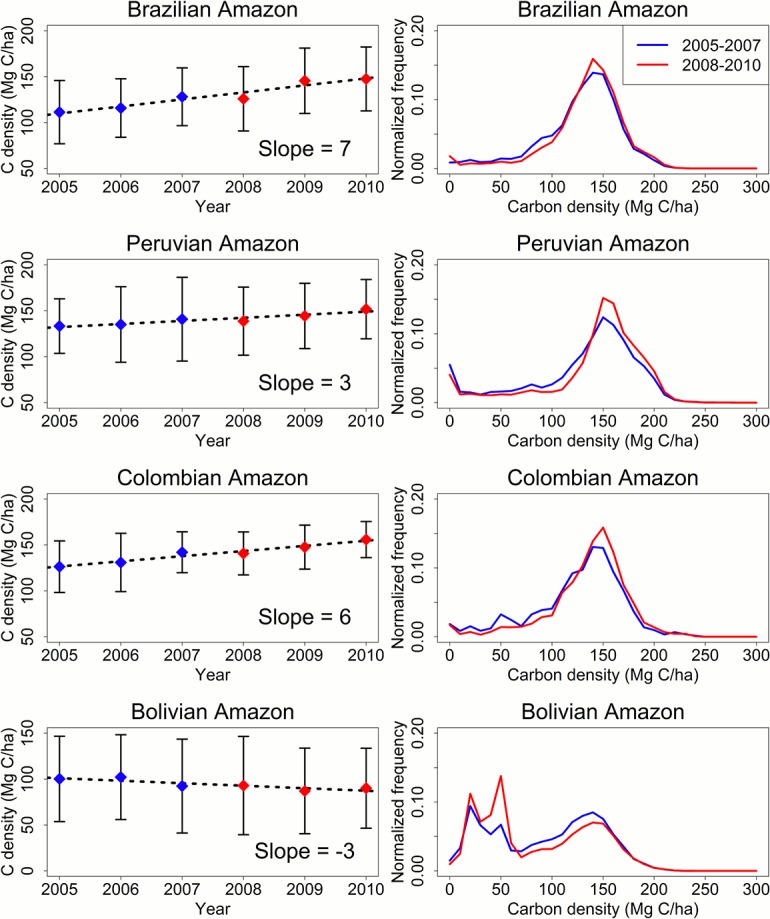
Carbon density of lost forests between 2005 and 2010. The left column shows trends in different regions over time. Blue and red diamond dots represent the mean value, with vertical bars representing ± one standard derivation. Dotted lines represent the linear fit of mean values. The right column shows distributions of carbon density, aggregated to two time periods 2005–2007 (blue lines) and 2008–2010 (red lines).

Calculated as the ratio of emission over deforestation area, the average carbon density of cleared forests or emission factors in the IPCC (Intergovernmental Panel on Climate Change) terminology [11] also varied over time and differed substantially among different regions. Here we focus on the 2005–2010 period in discussing the emission factors, because some of the Lidar (Light Detection and Ranging) data used to derive the carbon density map were acquired in 2003–2004 [23], and hence may not allow accurate calculation of the emission factors for clearing occurred in or before 2004 [49, 50]. The Colombian Amazon and the Peruvian Amazon had the highest emission factors, averaging at 141 Mg C∙ha^-1^ between 2005 and 2010, followed by Brazil (129 Mg C∙ha^-1^) and Bolivia (94 Mg C∙ha^-1^). The 2005–2010 basin-wide average emission factor was 130 Mg C∙ha^-1^. When calculated annually, these emission factors had different trends in different countries (Fig 6). From 2005 to 2010, statistically significant increasing trends (p < 0.05) were found in Colombia, Peru and Brazil, which had slope values of 6 Mg C∙ha^-1^ yr^-1^ (Colombia), 3 Mg C∙ha^-1^ yr^-1^ (Peru), and 7 Mg C∙ha^-1^ yr^-1^ (Brazil). Bolivia had an opposite trend (p < 0.001) with a slope of -3 Mg C∙ha^-1^ yr^-1^. The basin-wide slope was 7 Mg C∙ha^-1^ yr^-1^ (p < 0.001).

These trends resulted from changes in the carbon density of the cleared forests. Brazil, Peru, and Colombia had slightly higher proportions of high carbon density forests that were cleared between 2008 and 2010 than those cleared before 2008, while their proportions of low carbon density forests cleared between 2008 and 2010 were lower than those cleared before 2008 (Fig 6). These results indicate that deforestation has been progressively encroaching into higher-biomass forests in the Amazon interior [51–53]. In Brazil this was probably due to the scarcity of available forests in the “arc of deforestation” (i.e. the southeastern Amazon edge) after more than 40 years of continuous clearing [51]. The trend in Peru might be partially caused by the recent rapid expansion of gold mining in high-biomass forests in southern Peru [41]. In Bolivia, the relative proportions of low carbon density forests (i.e., < 60 Mg C∙ha^-1^) cleared after 2007 were higher than those cleared between 2005 and 2007, indicating increasing clearing of the low-biomass Chaco forests in this country.

## Discussion

### Effectiveness of MODIS VCF Products for Deforestation Monitoring

A key component of REDD+ is a credible system for measuring, reporting and verifying (MRV) changes in forest area and carbon stock [54]. In general, medium resolution data acquired by Landsat or Landsat-class satellites e.g. SPOT (Satellite Pour l'Observation de la Terre) are deemed necessary for deriving reliable estimates of forest change [55]. However, many areas have frequent cloud cover and often do not have enough cloud-free images for forest change assessment at the required temporal intervals (e.g., annual) [16, 56–58]. Some developing countries currently do not even have the minimum capacity for establishing Landsat-based annual forest monitoring systems for REDD+ MRV [54, 55]. Since the MODIS VCF based approach for quantifying deforestation and carbon emissions can produce results that are comparable to those derived using Landsat-based approaches, it may serve as a credible alternative when a Landsat-based MRV system is not available or not feasible due to lack of adequate cloud-free Landsat images. From an operational perspective, it is suggested that a nested framework consisted of multi-resolution satellite data as well as in-situ observations should be adopted in order to effectively and accurately monitor changes in forest cover and carbon stock in developing countries [59]. The MODIS VCF approach presented in this study may be used as the top layer (i.e. global, coarse-resolution data) of the framework.

Currently, MODIS VCF is produced annually for all land areas of the globe [45]. However, MODIS images the entire globe on a daily basis and produces near cloud-free global datasets at monthly or seasonal intervals. Therefore, it may allow development of VCF products at sub-annual intervals. Should such sub-annual VCF products become available, the approach developed through this study may allow forest monitoring at sub-annual intervals. This approach likely will be applicable in the foreseeable future, as MODIS-like data will be acquired continuously through the Visible Infrared Imaging Radiometer Suite (VIIRS), which is onboard the Suomi National Polar-orbiting Partnership (S-NPP) satellite launched in 2011 and will be deployed on the Joint Polar Satellite System (JPSS), NOAA’s (National Oceanic and Atmospheric Administration) next generation polar-orbiting operational environmental satellite system.

### Implications of Annual Emission Estimates for REDD+ Baseline Setting

Being able to derive deforestation and emission estimates annually or more frequently may have a significant impact on REDD+ policy, although the specific contribution is unclear until the REDD+ agenda is precisely defined in terms of implementation. Among numerous challenges confronting REDD+, defining the reference emission level (REL) or baseline is one of the most urgent because REL is a crucial input in determining the amount of financial credits generated from REDD+ [60–63]. A number of proposals have been submitted to UNFCCC (United Nations Framework Convention on Climate Change) for baseline setting, including the combined incentives approach [64], the compensated reductions approach [65], the corridor approach [36], the Joint Research Centre approach [66], the stock flow approach [67] and the Terrestrial Carbon Group approach [68]. A common component of baseline in these proposed methods is the historical emission rate, which refers to the mean emission rate over a moderately long time period (e.g. 5–10 years). While the scientific community has yet to reach a consensus on the methods for setting REL [60], we argue that any method selected should be flexible enough to account for the different temporal dynamics of deforestation emissions in different countries.

**Fig 7:**
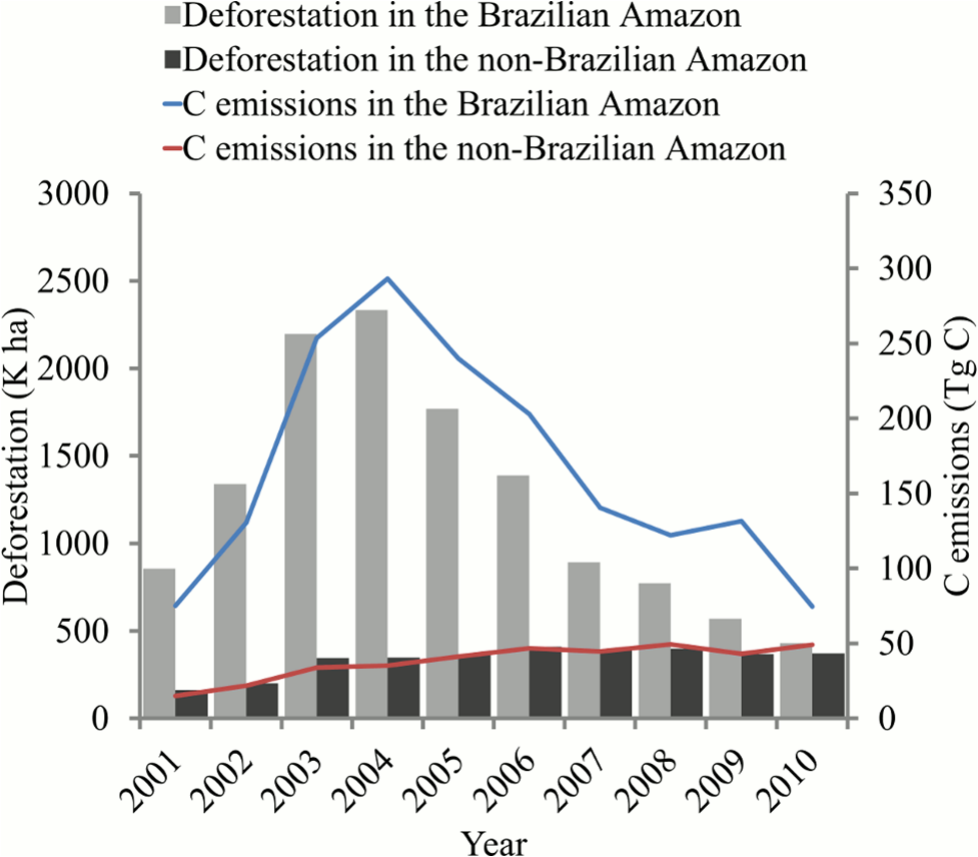
Annual deforestation and associated carbon emissions in the Brazilian and non-Brazilian Amazon.

The temporal dynamics of emissions observed in this study indicate that determining consistent REL for REDD+ may often be difficult. A marked example being that the Brazilian Amazon and the non-Brazilian Amazon have experienced generally opposite trends over the last decade (Fig 7). Emissions from deforestation also present various patterns of inter-annual variability at different spatial and temporal scales (Fig 3). High inter-annual variability can create particular challenges in a REDD+ payment system, as funding flows would vary greatly from year to year with the REL fixed over several years. Hence, trends and inter-annual variability within a specific time frame are highly relevant metrics for a REL formula. In practice, future reduction in deforestation under specific mitigation projects should be treated differently when the reduction is within or exceeds the natural variability.

Additional complexities to REDD+ REL setting are related to forest degradation (the second “D”) and forest regrowth (the “+”), which are not assessed in this study. It has been suggested that selective logging could contribute as much as 25% more carbon emissions in the Brazilian Amazon than accounting for deforestation alone [69]. Tropical regrowth forests can offset as much as 50% of gross carbon emissions from deforestation [7, 70]. However, compared with the estimation of carbon emissions from deforestation, how to accurately quantify carbon fluxes from forest degradation and forest regrowth remains an open scientific question [53].

### Uncertainties in Carbon Emissions from Deforestation

It remains a major challenge to conduct a comprehensive assessment of uncertainties in carbon emission estimation [70, 71]. In this study we take into account the two largest sources of uncertainty in emission estimation―uncertainties in deforestation estimates and uncertainties in biomass estimates. Discussion on the relative contribution of deforestation data and biomass data as well as the scale of analysis to emission uncertainty can be found in previous studies [24, 52, 53, 72]. Our deforestation area estimates derived from MODIS VCF and Landsat sample are proved to have ±16% uncertainty. Due to the combined uncertainties from both datasets, the emission rates have an uncertainty range of ±38%. This suggests that one third of the emission uncertainties are inherited from the deforestation map and two thirds are from the biomass map. Compared with other remote sensing-based emission estimates, our uncertainty range is smaller than DeFries *et al*. (42–50%) [5] and Harris *et al*. (40%) [8], but larger than Achard *et al*. (27%) [4] and Achard *et al*. (33–36%) [16].

The factor that is not explicitly considered here but may potentially increase our uncertainty estimates is errors associated with the Landsat reference data (i.e. errors in PRODES due to cloud or misclassification). If we were to estimate net carbon emissions, potential uncertainties would also include those associated with other forest dynamics such as degradation and regrowth, those associated with other significant carbon pools (i.e. dead wood, litter and soil) as well as those associated with the land cover dynamics on deforested land. Beside these factors, to reach a conceptually comprehensive estimate of carbon emissions from land cover and land use change as well as associated uncertainty, Houghton *et al*. summarize a list of land use processes that are often omitted in many or all existing studies, which includes forest management, agricultural management, fire management, land degradation, peatlands, wetlands and mangroves, human settlements and infrastructure, erosion/redeposition and woody encroachment [70].

### Risks of Future Deforestation in the Amazon

**Fig 8:**
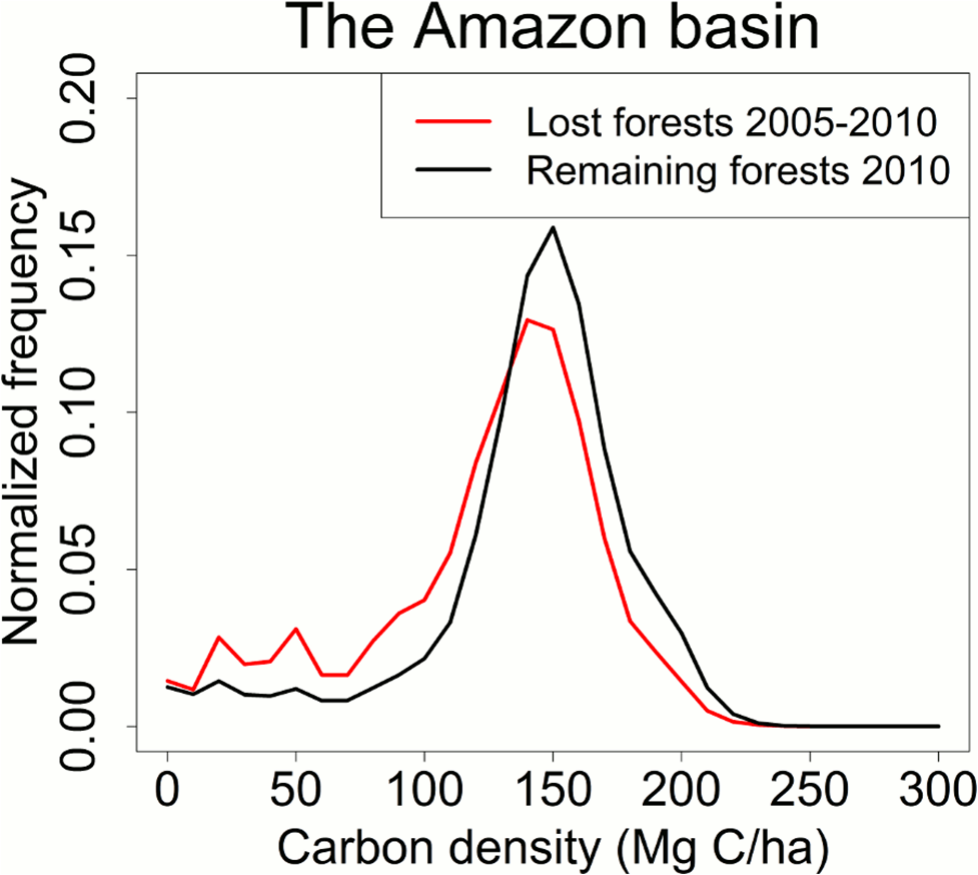
Carbon density of lost forests and remaining forests in 2010. Deforestation in the Amazon basin occurred in relatively lower biomass forests between 2005 and 2010.

Closed-canopy forests in the Amazon have high carbon stocks peaked around 150 Mg C∙ha^-1^ (Fig 8), but deforestation in tropical America is reported to have occurred in relatively lower-biomass lands between 2000 and 2005―the average carbon density of lost forests is 90 Mg C∙ha^-1^ by [8, 73] and 88 Mg C∙ha^-1^ by [6]. Our results reveal the same conclusion for the period of 2005–2010―the basin-wide average carbon density of remaining forests in year 2010 is 144 Mg C∙ha^-1^, ~11% higher than the average carbon density of cleared forests after 2005 (130 Mg C∙ha^-1^). Methodologically, this suggests that using a biome-level average biomass value in non-spatial carbon accounting models e.g. the bookkeeping model [71] or the IPCC Tier 1 and Tier 2 approaches [11] may overestimate emissions by more than 10%. It is also reported that deforestation has been encroaching into higher-biomass lands between 2001 and 2007 in the Brazilian Amazon [51, 53]. Our findings here show that the encroaching trend continues to year 2010. This trend would boost future carbon emissions from deforestation, if deforestation rates increase or even remain stable.

**Fig 9:**
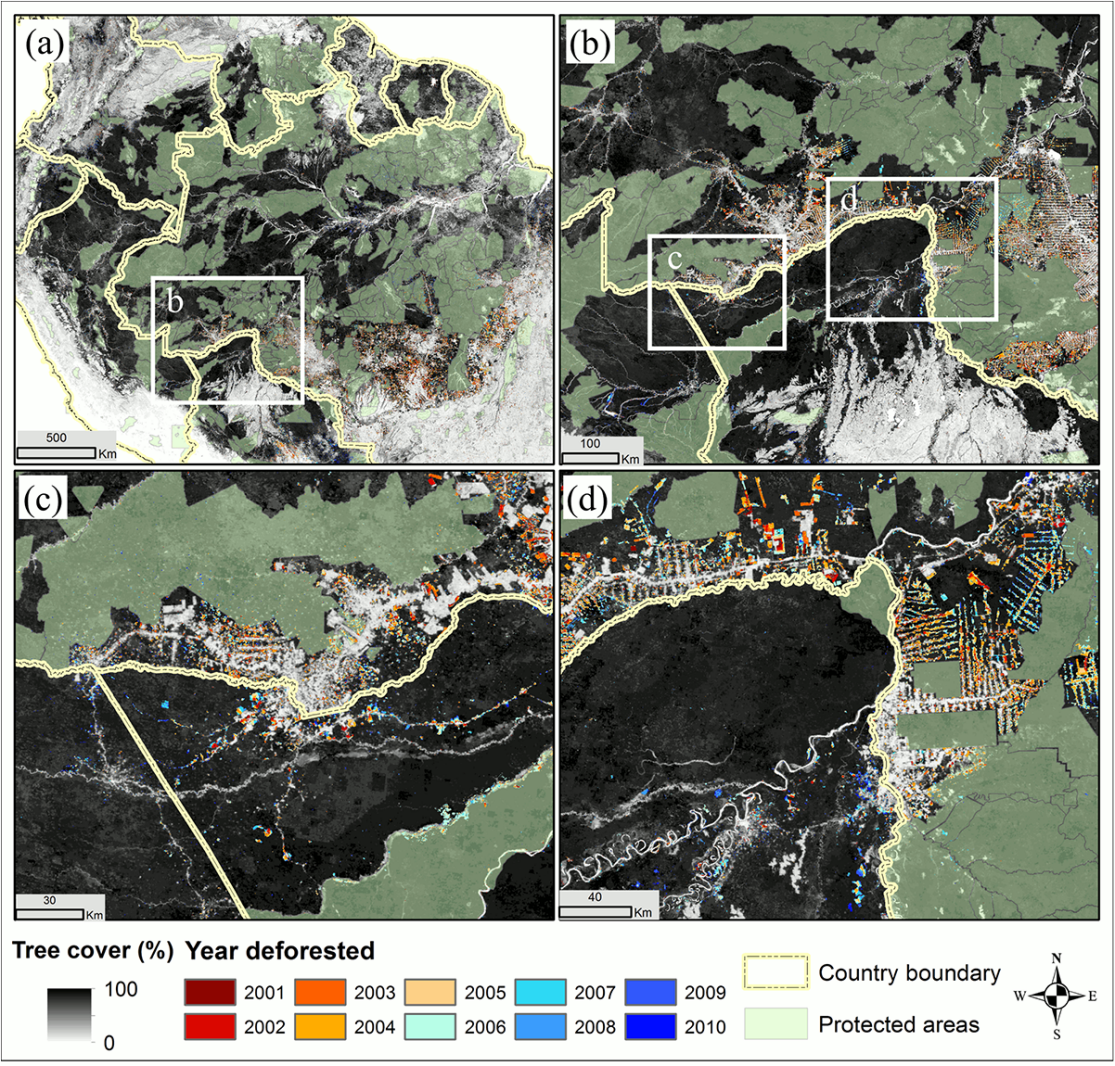
Forest, deforestation and protected areas in the Amazon basin. Four map layers are overlaying on each other in the order of (from top to bottom): country boundary, protected areas, deforestation year map and tree cover map in year 2000. (a) Overview of the entire basin. (b) A close-up in the Brazil/Bolivia/Peru tri-national border where forests on the Brazil side are either cleared or protected. (c) Further zoom-in over the city of Cobija, the capital of the Bolivian Pando Department. The inter-oceanic highway begins in this region. (d) Zoom-in over the city of Guayaramerin, where more deforestation is observed on the Bolivia side after year 2006.

Deforestation in the Brazilian Amazon has been attracting huge attention from the scientific community as well as the general public. However, ~40% of the Amazonian rainforests grow outside Brazil. Forests in western Amazon contain the highest live biomass as well as the richest biodiversity, including a large number of endemic and threatened species [37]. From 2000 to 2010, the Bolivian Amazon, the Peruvian Amazon and the Colombian Amazon all experienced an increase in deforestation when deforestation in the Brazilian Amazon plunged. Whether the low deforestation rate in Brazil can be sustained or not is yet to be determined. Questions can also be asked, for example, are these countries at different phases of forest transition [74]? Or, will the rising deforestation in the non-Brazilian Amazon continue? Relatively higher deforestation rates were found on the Bolivia and Peru side along the Brazil/Bolivia/Peru tri-national border after 2007 when forests on the Brazil side have been either cleared or designated as protected areas (Fig 9). Because accessibility to a road is often closely related to deforestation [75], the risks that the “arc of deforestation” may expand from Brazil to the most bio-diverse, most carbon-rich, yet mostly unprotected rainforests in Northern Bolivia and Southern Peru following the recent completion of the interoceanic highway appear high [76].

## Conclusions

We have demonstrated the effective use of satellite data for estimating deforestation and associated carbon emissions on a year-to-year basis. The increased temporal resolution is useful for understanding the global atmospheric CO_2_ variability and also provides important information for emerging policies such as REDD+. We found that carbon emissions from deforestation varied considerably not only among different regions but also from year to year. Largely driven by Brazil’s efforts to halt deforestation in recent years [32], deforestation rates over the Brazilian Amazon and the entire basin declined significantly in the second half of the last decade, which resulted in greatly reduced carbon emissions. An opposite emission trend was observed in the non-Brazilian Amazon; this consisted of various inter-annual variability in the Bolivian Amazon, the Colombian Amazon and the Peruvian Amazon. Furthermore, forests of higher-biomass accounted for an increasing portion of the cleared area. This trend plus the fact that remaining forests have higher biomass than previously cleared forests poses a new challenge for projecting carbon fluxes of future deforestation. Using a national or regional average carbon density value in non-spatial carbon accounting models may overestimate emissions by more than 10%. Spatially explicit and temporally consistent monitoring of forest cover and carbon stocks, like those used in this study, are needed to address this problem. Since our method can be implemented using long-term operational meteorological satellite data, continuity of this study is expected in the foreseeable future. Ultimately, a comprehensive understanding of the complex and dynamic drivers of deforestation is needed to devise effective policies to mitigate global deforestation [77].

## Materials and Methods

### Yearly MODIS VCF Tree Cover Dataset

Forest change products over the study area were derived from the yearly MODIS VCF tree cover data. MODIS VCF characterizes the Earth’s land surface as percent tree cover at a spatial resolution of 250 m. The yearly VCF products for the years between 2000 and 2010 were used in this study [45]. These products were derived following an established method described in [78]. Specifically, a bagged regression tree model was trained using a large Landsat-based reference sample and annual phenological metrics composited from the 16-day MODIS surface reflectance. The regression tree model was applied to annual MODIS metrics to predict percent tree cover per pixel per year. The latest version of this product i.e. MODIS Collection 5 VCF is downloadable at http://glcf.umd.edu/data/vcf/. Validated against measurements of tree cover from small-footprint Lidar data in four sites across three different forest biomes, errors in this global product were estimated to range from 7 to 21% in terms of root-mean-square-error (RMSE) [19]. It should be emphasized that the annual VCF product was generated based on atmospherically corrected surface reflectance―a unified physical value that enabled spatial consistency for global characterization of tree cover as well as temporal consistency for change analysis.

### Tracking Continuous Changes in Tree Cover

We designed a novel method to characterize deforestation from time series of tree cover layers. A detailed description of this method and validation of the resulting forest disturbance product was reported in [79]. We provide here a brief summary of the method as follows. A deforestation event typically has three distinctive stages over time: a pre-change stage when the VCF value is stable, a change stage when the VCF value declines sharply or gradually, and a post-change stage when the VCF value stays low until recovery occurs. This three-stage dynamic process is modeled using a logistic function:

$$f=\frac{a}{1+b^{\left(c-x\right)}}+d$$ (1)

**Fig 10:**
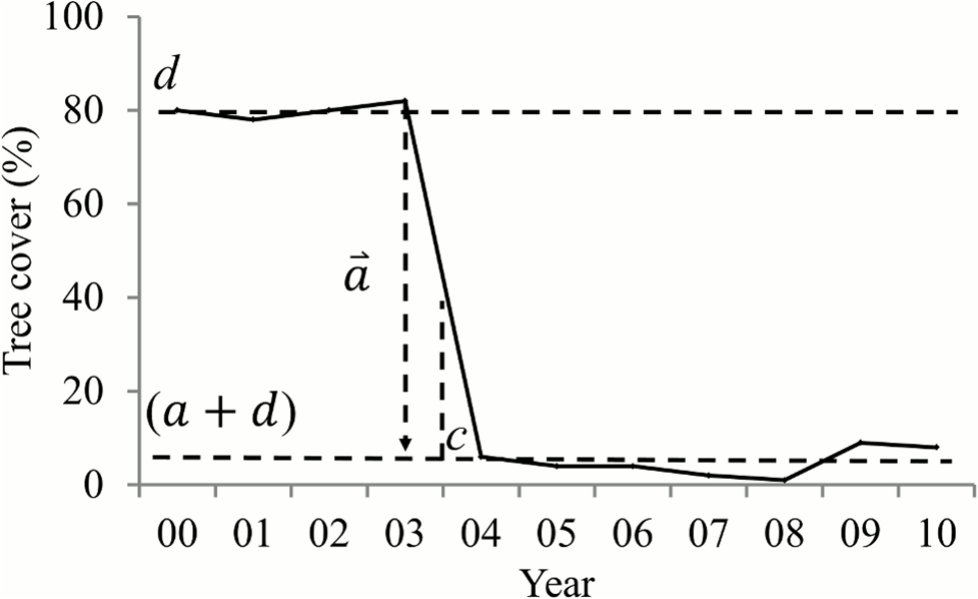
Structural parameters to characterize a deforestation event.

Where, *f* is tree cover in year *x*; parameter *a* defines the magnitude of change where negative values indicate deforestation; *b* controls change rate where large values indicate abrupt change and small values indicate gradual change; *c* denotes the inflection time when the change occurs, and *d* represents the pre-change value (Fig 10). The post-change VCF value is thus given by (*a+d*).

The four parameters were initialized and iteratively updated to reach a least-squared fit and the fitted curve was evaluated using a standard F-test based on the chi-square value of the fit. Pixels that had negative *a* values and passed the goodness-of-fit test (p < 0.01) were labelled as deforestation indicator. If multiple disturbances occurred in the 11-year period, a single logistic fit could not capture these change events. Hence we adjusted the procedure using a 5-year moving window to fit segments of the temporal VCF vector. The window size of 5-year was chosen because (1) this ensured the minimum number of observations required to estimate four parameters and (2) multiple deforestation events were highly unlikely to occur within five years. Goodness-of-fit test was also applied to each fitted segment to identify candidate change pixels. We implemented this curve fitting procedure for every MODIS pixel (n ~ = 111 million) across the study region and labelled every candidate deforestation pixel.

### Calibrating MODIS Deforestation Rate Using Landsat Data

Land cover change products derived using MODIS or coarser resolution data are typically considered indicator products and need to be calibrated using Landsat-based products to produce more accurate change estimates [17, 18, 80]. We calibrated the MODIS deforestation indicators using a large, systematically selected sample of Landsat images. The calibration was carried out in three steps: (1) deriving deforestation from Landsat images (2) adjusting for the difference between MODIS and Landsat acquisition dates and (3) searching for optimal thresholds to match MODIS-based deforestation rate with Landsat-based rate.

**Fig 11:**
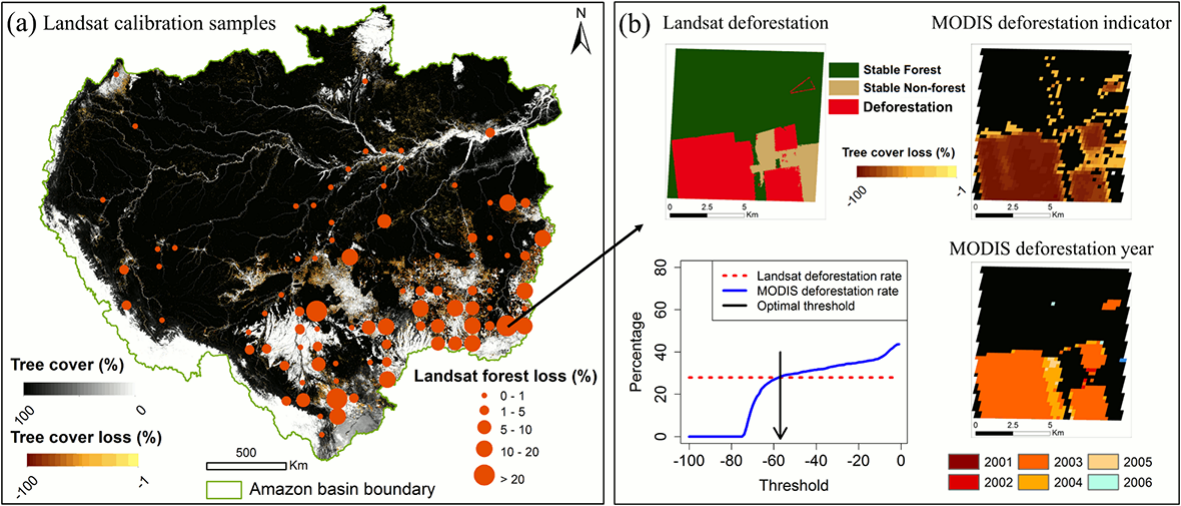
Calibrating MODIS indicators to derive accurate deforestation rates. (a) Percent tree cover in year 2000, model-fitted tree cover loss (deforestation indicator) between 2000 and 2010, and the location and deforestation rates of Landsat sample blocks. (b) An example of Landsat deforestation, MODIS deforestation indicator, the optimal threshold and the resulting MODIS deforestation year map.

The Landsat data used in calibration were selected from the Global Land Survey (GLS) collections circa 2000 and 2005 [81]. The GLS Landsat images were first converted to surface reflectance and then to percent tree cover at 30 m resolution [19]. Landsat deforestation was characterized based on the 2000–2005 tree cover layers using a post-classification probabilistic change detection algorithm [82]. Water, cloud and shadow pixels were identified using methods reported in [83, 84]. We used the systematic sampling scheme of FAO’s global remote sensing survey to select samples for calibration. Each sample site is 10 km × 10 km at each 1-degree intersection of latitude and longitude [85]. For every sample polygon, both Landsat deforestation and MODIS fitted layers were clipped to the spatial extend of the polygon. Landsat samples contaminated with more than 10% cloud and cloud shadow pixels in either date were removed. As a result, a total of 89 samples were collected in the study area (Fig 11).

The acquisition dates of the GLS images used in this study range from 2000-01-26 to 2002-11-13 for the 2000 epoch and from 2004-01-02 to 2007-10-29 for the 2005 epoch, which also vary from tile to tile. To resolve the time difference between Landsat and MODIS samples, we linearly normalized the Landsat-based deforestation rates to a common date of 30 June for the particular Landsat acquisition year. Samples contaminated by remaining cloud and cloud shadow (< 10%) were also linearly adjusted, assuming change rates in the cloudy area were the same as cloud-free area of the sample site [16, 85, 86]. Since parameter *c* indicates the timing of deforestation, we use the fitted parameter *c* value to select MODIS pixels where deforestation likely occurred within the two Landsat dates.

The third step was to search an optimal threshold for parameter *a*, such that the MODIS-derived deforestation rate matched with the Landsat-derived deforestation rate (Fig 11B). This threshold was determined for every sample block and the mean value of a country was applied to all MODIS pixels within the country to label deforestation. Due to the much larger size of Brazil, each of its states was treated as a “country” for the purpose of this calibration. For “countries” that do not have enough samples, we applied a basin-wide average threshold. It should be noted that the use of Landsat sample here was different from previous studies [16, 85, 86], in which deforestation rates were determined entirely based on the Landsat sample, whereas we used the sample as representative training to derive a threshold such that our MODIS rates matched Landsat rates at the block level. In most cases, Landsat blocks functioned mainly as a reference to clean edge pixels and salt-and-pepper noises at the MODIS resolution (Fig 11B). Our final deforestation rates were derived from the wall-to-wall MODIS data.

### Combining Deforestation and Biomass Maps to Estimate Carbon Emissions

The forest carbon density map (Fig 2C) used in emissions estimate was derived from multi-sources satellite data and *in situ* forest inventory plots [23]. Over one million laser shorts were used to derive forest structure metrics, which were related to above-ground biomass, below-ground biomass and carbon density (50% of total biomass) by applying field-calibrated allometric equations. The spatially contiguous carbon density and uncertainty maps were produced by integrating Lidar data with MODIS, shuttle radar topography mission data as well as quick scatterometer data at 1 km resolution.

We followed the standard methodology described in [8, 16] and the IPCC guidelines [11] in calculating gross carbon emissions from deforestation assuming immediate carbon release at forest clearing. To resolve the resolution discrepancy and reduce the geolocation mismatch between the deforestation map and the carbon density map, we aggregated both maps to 5 km resolution and calculated the lost carbon for every 5 km grid (in Mg C). We then summarized all 5 km grids over the entire study area to calculate carbon emissions for every year between 2000 and 2010.

It is important to re-emphasize here that our study specifically focuses on quantifying the committed loss of above and below ground biomass as a result of deforestation. Estimating net carbon emissions from forest change requires taking into account carbon fluxes from other forest dynamics such as forest degradation and regrowth of secondary forest as well as changes in other significant terrestrial carbon pools (i.e. dead wood, litter and soil) [53,71].

### Uncertainty Estimates

Uncertainties in quantifying carbon fluxes from deforestation arise from two major sources: errors in both deforestation and biomass estimates [71, 72]. Here we first characterize errors in the MODIS-based deforestation rates relative to those derived from Landsat data (considered as “truth”) and then combine this error with the carbon error map to analyze uncertainties in emission estimates using an established error propagation model [11, 87].

Independent deforestation maps produced by the PRODES project were used to evaluate the overall accuracy of the MODIS deforestation. We downloaded a total of 50 Landsat tiles completely covering the Amazon portion of Mato Grosso and Rondonia as reference data. PRODES maps in these areas were chosen because (1) they were generated by local experts using Landsat images and were found highly reliable [46, 48, 88, 89]; (2) Mato Grosso and Rondonia had high deforestation rates, accounting for about 50% deforestation of the study region; (3) Mato Grosso is dominated by large-scale extensive forest clearing for mechanized agriculture, which is also representative of Para, while Rondonia is famous for its small-scale “fishbone” pattern deforestation for frontier settlements, which is also found in Acre, Amazonas and Roraima [33, 34, 90, 91]; and (4) because these two states are located on the southeastern rim of the Amazon basin, they are less affected by cloud as compared with other Brazilian states within the basin. It should be noted that PRODES has a minimum mapping unit of 6.25 ha, close to the MODIS resolution but PRODES does not capture the clearing of secondary forests, which is included in our map [88, 89]. An annual comparison of MODIS and PRODES deforestation results over the entire Brazilian Amazon was presented in the previous sections of this paper. Here we focus on error estimation in a spatially explicit way.

**Fig 12:**
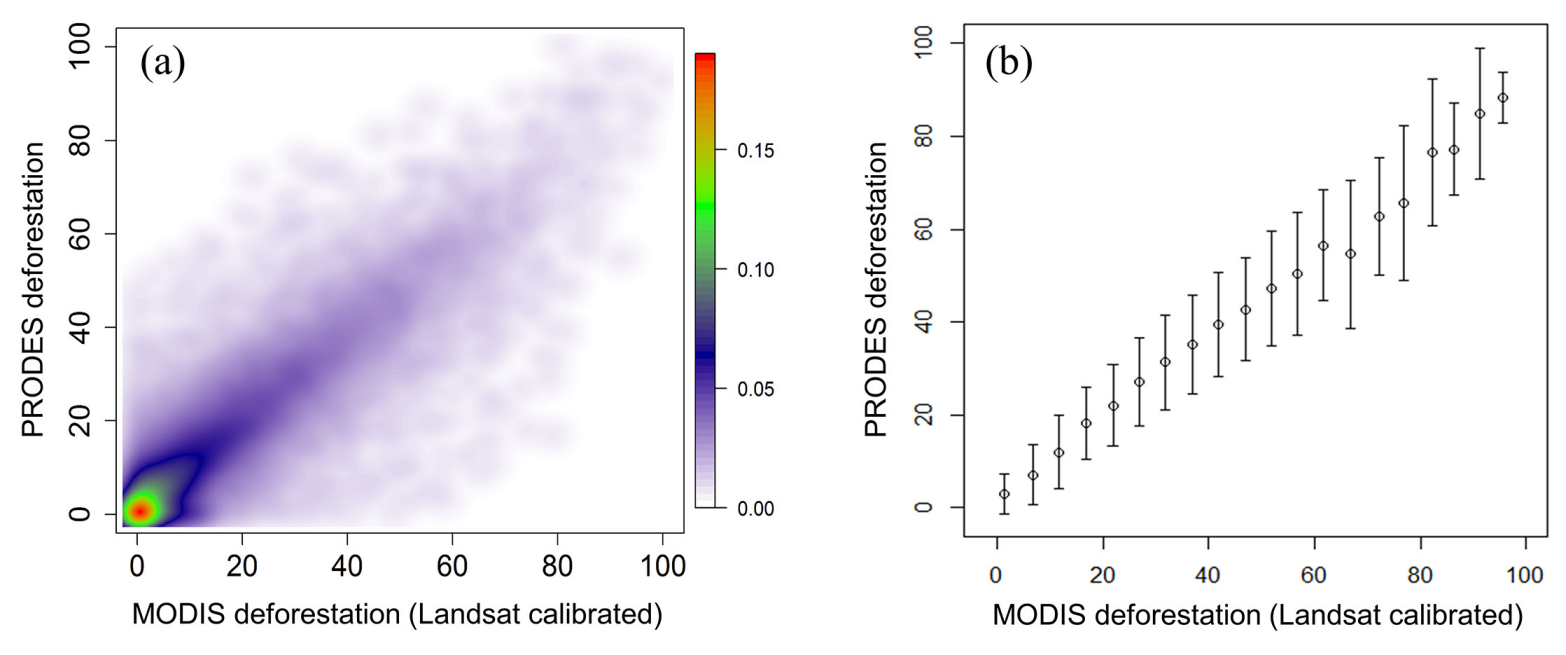
Estimating errors in MODIS-derived deforestation rates with PRODES as reference. (a) Density scatter plot with colours representing point density (n = 14,322). (b) Error bars represent ± one standard derivation of Landsat-derived deforestation rates for each deforestation level (bin = 5%).

The 30 m PRODES maps with pixels labelled as deforestation between 2000 and 2010 were aggregated to 5 km resolution to derive an 11-year deforestation rates per grid. The MODIS deforestation map was also aggregated to 5 km resolution to derive an overall deforestation rate between 2000 and 2010 (Fig 12). We then stratified the points based on MODIS rates (bin = 5%) and calculated the standard derivations of corresponding reference rates for each deforestation level (Fig 12B). These standard errors were applied to every 5 km grid of the entire study area. Although regional variations exist, the standard errors derived using this independent, large reference sample provide a reasonable error bound for deforestation estimates from MODIS.

We generated error bound for emission estimates using the error propagation model defined in the following equation. Both deforestation and carbon density error terms are expressed in terms of percentage of relative error and assuming they are independent [11, 87], the propagation model is given by:

$$\varepsilon_{carbon\;emission}={\left(\varepsilon_{deforestation}^{2}+\varepsilon_{carbon\;density}^{2}\right)}^{1/2}$$ (2)

where *ε*_*carbon emission*_ refers to errors in emission estimates; *ε*_*deforestation*_ represents errors in deforestation estimates and *ε*_*carbon density*_ represents errors in carbon density estimates. Errors in carbon density estimates were quantified from four components including (1) measurement error associated with tree height estimation from Lidar data, (2) allometric error associated with biomass estimation from tree height, (3) sampling error associated with the representativeness of sample plots and the spatial variation of biomass within a 1 km pixel and (4) prediction error of the machine learning model [23]. The error propagation model was applied to every 5 km grid. We then calculated the upper bound of emission for every grid by adding this error term to the mean estimate as well as the lower bound by subtracting this error term from the mean estimate. Basin-wide upper emission estimate and lower emission estimate was then derived by summarizing all 5 km grids of upper estimate and lower estimate within the study region, respectively.
